# What Is Your Choice for Androgen Deprivation Therapy in Metastatic Prostate Carcinoma: Surgical or Medical?

**DOI:** 10.5152/tud.2022.22076

**Published:** 2022-07-01

**Authors:** Huseyin Salih Semiz, Erdem Kisa, Eda Caliskan Yildirim, Elif Atag, Mehmet Emin Arayici, Talha Muezzinoglu, Aziz Karaoglu

**Affiliations:** 1 Department of Medical Oncology, Dokuz Eylül University, Institute of Oncology, İzmir, Turkey; 2 Department of Urology, Tepecik Education and Research Hospital, İzmir, Turkey; 3 Division of Medical Oncology, Department of Internal Medicine, Dokuz Eylül University Faculty of Medicine, İzmir, Turkey; 4 Department of Medical Oncology, Haydarpaşa Education and Research Hospital, İstanbul, Turkey; 5 Department of Preventive Oncology, Dokuz Eylül University, Institute of Health Sciences, İzmir, Turkey; 6 Department of Urology, Manisa Celal Bayar University Faculty of Medicine, Manisa, Turkey

**Keywords:** LHRH analogs, prostate carcinoma, androgen deprivation therapy, orchiectomy

## Abstract

**Objective:**

**:** At the time of diagnosis, approximately 16.5% of prostate cancer patients are metastatic. The main framework of metastatic prostate cancer treatment is androgen deprivation therapy, which is performed surgically or medically. The aim of this study is to evaluate the attitudes of medical oncologists and urologists about orchiectomy as androgen deprivation therapy.

**Material and Methods:**

A total of 387 physicians working in the Departments of Urology (n = 217) and Medical Oncology (n = 170) were included in this descriptive study. Data were collected through an electronic survey.

**Results:**

Only 7.5% of participants indicated that they offered surgical castration to their patients. Urologists preferred surgical castration more than oncologists for the treatment of metastatic castration-sensitive prostate carcinoma (*P* = .003). The reasons why medical oncologists preferred surgical castration less are that it is an invasive procedure, has risk of morbidity and mortality, high cost of hospitalization, and may cause deterioration of the patient's body image (*P* < .05).

**Conclusion:**

This study showed that physicians were less likely to perform orchiectomy as an androgen deprivation therapy. Although the most important reason for this is the patient preference, the biased presentation of treatment options to patients, the lack of knowledge of physicians about orchiectomy, and the effect of the pharmaceutical industry should also be kept in mind.

**Main Points**

- Orchiectomy is one of the standard treatment modalities in men with metastatic prostate cancer. However, physicians usually do not prefer orchiectomy, which is an inexpensive and highly effective form of androgen deprivation therapy (ADT).
- This survey revealed that both urologists and medical oncologists were less likely to prefer orchiectomy as an ADT. However, urologists preferred surgical castration more than medical oncologists.
- Physicians in academic centers were more likely to prefer orchiectomy than medical ADT.
- The reasons why medical oncologists preferred surgical castration less are that it is an invasive procedure, has risk of morbidity and mortality, high cost of hospitalization, and may cause deterioration of the patient’s body image.

## Introduction

Prostate carcinoma is the second most common cancer in men in Turkey.^1^ At the time of diagnosis, approximately 16.5% of patients have metastatic disease.^2^ Five-year survival rate with metastatic disease is around 30%.^3^ The main method for the treatment of metastatic prostate cancer (mPCa) is testosterone-suppressive therapies, also called androgen deprivation therapy (ADT). These treatments can be divided into surgical castration (orchiectomy) and medical castration. Luteinizing hormone-releasing hormone (LHRH) analogs are used most frequently for medical ADT.^4^ Recently, clinical studies were conducted with LHRH antagonists, and impressive results were obtained.^5^ Surgical castration with bilateral orchiectomy is a relatively simple, cost-effective procedure, and it remains the standard treatment for mPCa in many countries.^6^ However, patients increasingly prefer medical ADT over orchiectomy. The most important reason for this seems to be that orchiectomy leads to deterioration in the body image of the patients.^7^ However, there is not enough information about the attitudes of medical oncologists and urologists regarding the choice of orchiectomy as ADT in patients with mPCa. In addition, the reasons why physicians do not prefer orchiectomy, which is an inexpensive and highly effective form of ADT, is an issue that needs to be examined.

The aim of this study was to evaluate the attitude and knowledge of medical oncologists and urologists about orchiectomy as an ADT.

## Material and Methods

The sample size was calculated with the OpenEpi open-access program. The plan was to reach at least 342 urologists and at least 259 medical oncologists at the level of 50% frequency in cases of unknown frequency, 5% worst accepted error rate, and 95% CI for 80% power. These numbers were calculated based on the knowledge that there are 3073 licensed urologists and 787 licensed medical oncologists working in Turkey. However, these numbers could not be reached during the period when the questionnaire was actively in circulation. A total of 387 Turkish physicians working in the Departments of Urology (n = 217) and Medical Oncology (n = 170) at university hospitals, public hospitals, training and research hospitals, and private hospitals were included in this descriptive study. The data were collected between August and December 2021. The study was approved by the Ethics Board of Tepecik Education and Research Hospital (Decision number 2021/08-12). Written informed consent was obtained from all participants who participated in this study.

Data were collected through a questionnaire (Appendix 1), which was created by the authors. The physicians were contacted online. Information was given about the study, and their consent was obtained. The questionnaire was filled out by the physicians on their own. The survey (Appendix 1) was constructed using Microsoft Forms® and distributed via email.

A pre-defined spreadsheet was created using Microsoft Excel® to systematically record the core qualitative and quantitative data obtained from the survey results. Demographics and current practices in the management of mPCa were among the data gathered. The responses were examined in a descriptive manner in order to spark debate over the use of surgical castration as ADT for prostate cancer.

The study variables in the research included descriptive characteristics of the participants (age, title, department, institution, clinic of specialty training, and years of study in the field of urology/medical oncology) and other answers given by the physicians. In accordance with the European Society of Medical Oncology (ESMO) Young Oncologist definition,^8^ the participants were divided into 2 groups as those under 40 years old and those over 40 years old. The answers given to the questionnaire were analyzed separately between these 2 groups for differences in responses to each question.

The data were analyzed with Statistical Package for Social Science version 24.0 (IBM SPSS Corp.; Armonk, NY, USA). For statistical analysis, descriptive statistics, the chi-square test, Fisher’s exact test, Kruskal–Wallis test, and Mann–Whitney *U* test were used as appropriate. The level of statistical significance was set as *P* < .05.

## Results

Responses were obtained from urologists (n = 217) and medical oncologists (n = 170 practicing in all provinces in Turkey. The mean age of the participants was 39.6 ± 9.8 years. About three-quarters (76.5%) of the physicians worked in university hospitals or training and research hospitals. The median number of patients examined in daily practice was determined as 40, while the median number of monthly mPCa patients was determined as 10. The descriptive characteristics of the participants are presented in Table 1.

The percentage of prostate cancer patients who are followed up and treated by a urologist was significantly higher compared to those followed by a medical oncologist (*P* < .001). However, the median number of mPCa patients seen in a month by oncologists (n = 22) is significantly higher than urologists (n = 7) (*P* < .001). When all physicians participating in the survey are evaluated together, the rate preferring orchiectomy as an ADT was determined as 7.5%. Urologists prefer surgical castration for the treatment of metastatic castration-sensitive prostate carcinoma more than medical oncologists (*P* = .003). They offer surgical castration as an option more often than medical oncologists for patients diagnosed with metastatic castration-sensitive prostate carcinoma (*P* < .001) (Table 2).

The answers of the participants regarding the reasons for their castration preferences are summarized in Table 3. Urologists reported that surgical castration is more effective (*P* = .010), less costly (*P* < .001), has less cardiovascular side effects (*P* < .001), less metabolic side effects (*P* = .002), is safer than medical ADT in terms of bone health (*P* = .032), and acts faster than medical ADT (*P* < .001) for patients with metastatic castration-sensitive prostate carcinoma. On the other hand, medical oncologists prefer surgical castration much less because it is an invasive procedure, there is a risk of morbidity and mortality caused by the operation, high cost of hospitalization, and deterioration of the patient's body image (*P* < .05).

The participants were divided into 3 groups according to the title as resident/fellow, academic, and specialist. On the basis of the title, there was no significant difference in terms of the percentage of patients cared for in daily practice, the percentage of patients whose follow-up and treatment are undertaken, and the percentage of mPCa patients seen in a month (*P* > .05). When the preference for medical androgen deprivation treatments in patients with metastatic castration-sensitive prostate carcinoma is evaluated, specialists and residents preferred leuprolide acetate every 3 months more than academics (*P* = .005). The rate of academics reporting that “surgical castration and medical ADT are equally effective” was higher than the others (*P* = .038). In addition, academics and specialists reported that “surgical castration is less costly than medical ADT” more than resident/fellow (*P* = .005). In addition, academics reported more than other participants that “surgical castration acts faster than medical ADT” (*P* = .036). When the reasons for not preferring surgical castration were reviewed in terms of title, no significant difference was found (*P* > .05). It was determined that the institution of study did not have a significant relationship with other parameters. In terms of surgical castration and medical ADT preferences, the training center (training and research hospital-university hospital) did not have any effect (*P* > .05). Academics recommend orchiectomy more compared to medical ADT in patients who progress to the metastatic castration-resistant prostate carcinoma stage while under medical ADT (*P* < .001). While 64.1% of physicians did not recommend medical ADT changes in patients receiving medical ADT and progressing to metastatic castration-resistant prostate carcinoma, 28.4% of physicians recommended this in their daily practice. Similarly, 62.5% of all participants did not recommend orchiectomy over medical ADT in patients who progress to metastatic castration-resistant prostate carcinoma stage while receiving medical ADT.

The answers to the castration questionnaire separated by age groups are summarized in Table 4. The proportion of patients with prostate cancer who were followed up and treated was significantly higher for physicians younger than 40 years of age compared to physicians aged 40 years and older (*P* = .002). Physicians aged 40 and over reported that “cardiovascular and metabolic side effects of surgical castration are higher than for medical ADT” compared with younger participants (*P* = .010, *P* = .020). When the reasons for not preferring surgical castration were compared according to age, no significant relationship was found for any of the sub-items (*P* > .05). Physicians aged 40 and over offered orchiectomy to patients less frequently (*P* = .042).

## Discussion

The reasons why patients do not prefer orchiectomy as ADT were investigated many times. The most common reasons are the deterioration of body image, the need for hospitalization and related difficulties, concerns about the risk of complications that may occur due to surgery, the irreversibility of the procedure, and a decrease in sexual desire and potency.^9^ Although patients’ opinions about orchiectomy are known, what medical oncologists and urologists who are involved in the treatment and follow-up of mPCa patients think and tend to do about this subject are unknown. In this study, it was determined that the rate of urologists preferring orchiectomy as an ADT was higher than medical oncologists. In addition, we found that urologists approach orchiectomy more positively in terms of effectiveness and side effects than medical oncologists. In our study, physicians participating in the survey stated that medical ADT and surgical ADT are similar in terms of effectiveness, in line with the literature. When all participants are evaluated together, the rate of recommending orchiectomy as an ADT to patients was only 7.5%. In a survey conducted by Anderson and Rowe with Canadian urologists, the rate of recommending orchiectomy was 11%. In this study, similar to ours, the most common reason why orchiectomy was not preferred was the negative attitude of patients toward this treatment method. Other reasons included the permanence of the procedure, difficulty in finding an operating room, and morbidities caused by the surgery.^10^ In our study, the reasons why orchiectomy was not preferred, especially as reported by medical oncologists, included the invasiveness of the procedure, the risk of morbidity and mortality, the high cost of hospitalization, and the deterioration of the patient’s body image.

In accordance with the ESMO'’s definition of a young oncologist, when all participants were divided into 2 groups as under 40 years old and over 40 years old, it was concluded that young physicians recommended orchiectomy more as an ADT, but the reason for this was not clearly revealed. In a study by Garje et al^11^, physicians working in academic centers preferred orchiectomy less than medical ADT. However, the results obtained in our study show that, contrary to this study, physicians in academic centers prefer orchiectomy more than medical ADT. This may be due to the working principles and budgets of academic centers in different countries. From an economic perspective, it is obvious that orchiectomy is much more advantageous than medical ADT.^12^ When the answers of participants on this subject were examined, there was a high level of awareness in our research. Considering the studies comparing the side effects of medical ADT and surgical ADT, surgical ADT seems to be safer in terms of cardiovascular health, metabolic parameters, and bone health.^13^ However, there are also some studies reporting that both ADTs are similar in terms of toxicity.^14^ The participants in our study declared that surgical ADT is safer than medical ADT in terms of all toxicities.

Considering the economic burdens brought by coronavirus disease (COVID-19) to countries,^15^ it would be extremely rational to choose treatment methods that are equally effective but less costly for the treatment of any disease.^16^ It is also suggested in many international guidelines that treatment methods should be chosen which will bring patients to the hospital less frequently during the COVID-19 pandemic.^16^ Considering that urologists are the first group of physicians to encounter prostate carcinoma patients in clinical practice and surgical methods are the main duty of this physician group, this may explain why they prefer orchiectomy more than medical oncologists. Of course, the effect of the pharmaceutical industry on both physicians should be considered in this regard. Patients’ preferences may change if the physician managing the patient’s treatment objectively discusses all options, including orchiectomy, with the patient.^17^ Orchiectomy with new surgical techniques such as subcapsular orchiectomy has been shown to provide effective castration and less psychosocial side effects.^7^ In order to change the prejudices about orchiectomy, physicians giving primary treatment should be given training under the guidance of current literature. The prolongation of life expectancy of patients with mPCa and the widespread use of new generation hormonal agents should be kept in mind as the toxicities of these agents may be added to the toxicities of medical ADT and cause serious morbidity in the long term.^18^ At exactly this point, many studies showed that the long-term toxicities of orchiectomy are less than medical ADT. This long-term toxicity problem may lead to higher preference for orchiectomy in future years.

When treating patients with castration-sensitive prostate carcinoma with any medical ADT, the development of castration-resistant disease is almost inevitable. In this case, it is known that there are various treatment approaches such as switching to another medical ADT or performing an orchiectomy instead. There are some data suggesting that changing the current medical ADT may be beneficial.^19^ However, it is not known at this stage how orchiectomy instead of medical ADT will affect the course of the disease. In our study, 62.5% of the participants stated that they did not prefer orchiectomy at this stage when asked about this issue. However, 31.5% of the participants stated that they applied this approach despite the lack of data in the literature. In fact, this data show that some of the choice for orchiectomy is used for castration-resistant patients.

It does not seem possible today to say whether performing orchiectomy in the castration-resistant stage is an appropriate treatment approach. However, it will remain experimental to choose orchiectomy in castration-resistant prostate carcinoma without clinical studies (if possible) and clear data on this subject. This issue needs clarification.

Our study is important in that it represents a first in the literature. However, there are some limitations as well. Among these, the planned number of participants could not be reached when the study was designed, the reasons for not preferring orchiectomy were not fully clarified at some points, and a balanced distribution of the participants could not be obtained. Another limitation is that radiation oncologists who treat patients with prostate cancer were not included in this survey.

Orchiectomy is one of the standard treatment modalities in men with mPCa. However, it is preferred less than medical ADT for many reasons, such as patient preference, the way physicians present treatment options, insufficient information of patients, physicians’ lack of knowledge of the literature about orchiectomy, and the effect of the pharmaceutical industry. Our study is the first study on this subject which comparatively evaluates the ideas and attitudes of medical oncologists and urologists about orchiectomy. Further research in this area is of critical importance, and there is a growing need for this in the literature.

## Figures and Tables

**Table 1.** Distribution of the Participants According to Demographic Features, Academic Features, and Descriptive Features

Participants’s age (mean ± SD)	39.6 ± 9.8
<40 years	229 (59.2)
≥40 years	158 (40.8)
Number of patients seen in daily practice (median)	40
Number of metastatic prostate cancer patients per month (median)	10
Titles of participants	
Resident/fellow	88 (23.2)
Specialist	118 (31.3)
Academics	171 (45.4)
Experience in Urology/Medical Oncology (years), n(%)	168 (43.4)
0-5	82 (21.2)
6-10	61 (15.8)
11-15	16 (4.1)
16-20	29 (7.5)
21-25	31 (8)
>25	82 (21.2)
Percentages of prostate cancer patients per day, n(%)	
1-10	254 (65.6)
11-20	98 (25.3)
21-30	24 (6.2)
31-40	8 (2.1)
41-50	2 (0.5)
>50	1 (0.3)
Percentages of metastatic prostate cancer patients per month, n(%)	
1-10	68 (17.6)
11-20	62 (16)
21-30	47 (12.1)
31-40	33 (8.5)
41-50	42 (10.9)
>50	135 (34.9)
Primarily preferred androgen deprivation therapy, n(%)	
Medical ADT	358 (92.5)
Surgical castration	29 (7.5)
Presents surgical castration as an option for patients with metastatic castration-sensitive prostate carcinoma	
No	51 (13.2)
Rarely	116 (30.0)
Sometimes	131 (33.9)
Often	50 (12.9)
Always	39 (10.1)

SD, standard deviation; ADT, androgen deprivation therapy.

**Table 2.** Castration Survey Responses Separated by Department

	**Primarily Preferred Androgen Deprivation Therapy**			
**Groups**	**Surgical Castration (n, %)**	**Medical ADT (n, %)**		*P* **
Medical oncology	5 (2.9)	165 (97.1)		**.003**
Urology	24 (11.1)	193 (88.9)	
**Recommends a medical ADT change in patients receiving medical ADT who progress to the metastatic castration-resistant prostate carcinoma stage**				
Groups*	**Yes**	**No**	**No Idea**	*P* **
Medical oncology	35(20.6)	126(74.1)	9(5.3)	**.001**
Urology	77(35.5)	122(56.2)	18(8.3)
**Recommend orchiectomy instead of medical ADT for patients who have progressed to the metastatic castration-resistant prostate carcinoma stage while receiving medical ADT**				
Groups*	**Yes**	**No**	**No Idea**	*P* **
Medical oncology	35 (20.6)	126 (74.1)	9 (5.3)	<.001
Urology	87 (40.1)	116 (53.5)	14 (6.5)

^*^1, I do not prefer at all; 5, I prefer very often; ^**^Chi-square test. P < .05 was considered as statistically significant.

ADT, androgen deprivation therapy.

**Table 3.** Distribution of the Answers Given to the Castration Questionnaire

Reflections on Surgical Castration in Patients with Metastatic Castration-Sensitive Prostate Carcinoma^**^	1	2	3	4	5
Equal to medical ADT	5 (1.3)	39 (10.1)	36 (9.3)	167 (43.2)	140 (36.2)
More effective than medical ADT	34 (8.8)	119 (30.7)	86 (22.2)	85 (22.0)	63 (16.3)
Less costly than medical ADT	8 (2.1)	17 (4.4)	26 (6.7)	123 (31.8)	213 (55)
Cardiovascular side effects are less than medical ADT	9 (2.3)	63 (16.3)	67 (17.3)	134 (34.6)	114 (29.5)
Metabolic side effects (dyslipidemia, hyperglycemia, etc.) are less than medical ADT	10 (2.6)	59 (15.2)	84 (21.7)	132 (34.1)	102 (26.4)
Safer than medical ADT in terms of bone health	17 (4.4)	83 (21.4)	138 (35.7)	92 (23.8)	157 (14.7)
Acts faster than medical ADT	4 (1.0)	22 (5.7)	46 (11.9)	133 (34.4)	182 (47.0)
Reasons for not preferring surgical castration^**^	1	2	3	4	5
Patients do not prefer orchiectomy	10 (2.6)	14 (3.6)	29 (7.5)	206 (53.2)	218 (33.1)
Being an invasive procedure	28 (7.2)	74 (19.1)	31 (8.0)	187 (48.3)	67 (17.3)
Risk of morbidity and mortality that may be caused by the operation	48 (12.4)	116 (30.0)	57 (14.7)	127 (32.8)	39 (10.1)
High hospitalization costs	90 (23.3)	187 (48.3)	56 (14.5)	40 (10.3)	14 (3.6)
I think it is not as effective as medical ADT	175 (45.2)	144 (37.2)	45 (11.6)	16 (4.1)	7 (1.8)
May cause deterioration in the patient’s body image	18 (4.7)	29 (7.5)	56 (14.5)	204 (52.7)	80 (20.7)
Orchiectomy lacks sufficient level of scientific evidence	178 (46)	151 (39.0)	43 (11.1)	8(2.1)	7 (1.8)
More expected toxicities than medical ADT	149 (38.5)	169 (43.7)	47 (12.1)	19(4.9)	3 (0.8)

^*^1, I do not prefer at all; 5, I prefer very often;^**^1, strongly disagree; 5, strongly agree.

ADT, androgen deprivation therapy.

**Table 4.** Castration Survey Responses Separated by Age

**Primarily Preferred Androgen Deprivation Therapy**				
**Groups**	**Surgical Castration(n, %)**		**Medical ADT (n, %)**	*P* **
Under 40 years	18 (7.9)		211 (92.1)	.741
40 years and above	11 (7.0)		147 (93.0)
**Recommends a medical ADT change in patients receiving medical ADT who progress to the metastatic castration-resistant prostate carcinoma stage**				
Groups*	**Yes (n, %)**	**No (n, %)**	**No Idea (n, %)**	*P* **
Under 40 years	56 (24.5)	15 (65.9)	22 (9.6)	**.008**
40 years and above	56 (35.4)	97 (61.4)	5 (3.2)
**Recommends orchiectomy instead of medical ADT for patients who have progressed to the metastatic castration-resistant prostate carcinoma stage while receiving medical ADT**				
**Groups** *	**Yes (n, %)**	**No (n, %)**	**No Idea**	*P* **
Under 40 years	63 (27.5)	147 (64.2)	19 (8.3)	**.015**
40 years and above	59 (37.3)	95 (60.1)	4 (2.5)

^*^1, I do not prefer at all; 5, I prefer very often; ^**^Chi-square test. P < .05 was considered as statistically significant.

ADT, androgen deprivation therapy.

## References

[1] Sung H Ferlay J Siegel RL et al. Global cancer statistics 2020: GLOBOCAN estimates of incidence and mortality worldwide for 36 cancers in 185 countries. CA A Cancer J Clin. 2021;71(3):209 249. 10.3322/caac.21660) 33538338

[2] Zorlu F Zorlu R Divrik RT Eser S Yorukoglu K . Prostate cancer incidence in Turkey: an epideiological study. Asian Pac J Cancer Prev. 2014;15(21):9125 9130. 10.7314/apjcp.2014.15.21.9125) 25422189

[3] Howlader N Noone AM Krapcho M et al., eds. SEER Cancer Statistics Review. Bethesda, MD: National Cancer Institute. 1975-2018. https://seer.cancer.gov/csr/1975_2018/, based on November 2020 SEER data submission, posted to the SEER web site, April 2021.

[4] Teo MY Rathkopf DE Kantoff P . Treatment of advanced prostate cancer. Annu Rev Med. 2019;70:479 499. 10.1146/annurev-med-051517-011947) 30691365 PMC6441973

[5] Crawford ED Hou AH ed. The role of LHRH antagonists in the treatment of prostate cancer. Oncology (Williston Park). 2009;23(7):626 630. 19626830

[6] Holm HV Dahl AA Klepp OH Fosså SD . Modern treatment of metastatic prostate cancer [Moderne behandling av prostatakreft med fjernmetastaser]. Tidsskriftet Den Norske Legeforening. Tidsskr Nor Laegeforen. 2017;137(11):803 805. 10.4045/tidsskr.16.0265) 28597635

[7] Selvi I Basar H . Subcapsular orchiectomy versus total orchiectomy and LHRH analogue in the treatment of hormone-sensitive metastatic prostate cancer: a different perspective in evaluation of the psychosocial effects. Support Care Cancer. 2020;28(9):4313 4326. 10.1007/s00520-019-05266-2) 31912363

[8] Esmo Young Oncologists Committee Web S ite. Available at: https://www.esmo.org/about-esmo/organisational-structure/young-oncologists-committee. Accessed 18 January 2022.

[9] Rud O Peter J Kheyri R et al. Subcapsular orchiectomy in the primary therapy of patients with bone metastasis in advanced prostate cancer: an anachronistic intervention? Adv Urol. 2012;2012:190624. 10.1155/2012/190624) PMC3172971 21922019

[10] Anderson PT Rowe NE . Current attitudes of Canadian urologists towards surgicalcastration in the treatment of prostate cancer. Can Urologicial Assoc J. 2020. 10.5489/cuaj.6834) PMC8095285 33119504

[11] Garje R Chennamadhavuni A Mott SL et al. Utilization and outcomes of surgical castration in comparison to medical castration in metastatic prostate cancer. Clin Genitourin Cancer. 2020;18(2):e157 e166. 10.1016/j.clgc.2019.09.020) 31956009 PMC7190190

[12] Rohde V Grabein K Hessel F Siebert U Wasem J . Orchiectomy versus medical therapy with LH-RH analogues for the treatment of advanced prostatic carcinoma. GMS Health Technol Assess. 2006;2:Doc13. 21289964 PMC3011358

[13] Sun M Choueiri TK Hamnvik OP et al. Comparison of gonadotropin-releasing hormone agonists and orchiectomy: effects of androgen-deprivation therapy. JAMA Oncol. 2016;2(4):500 507. 10.1001/jamaoncol.2015.4917) 26720632

[14] Chen DY See LC Liu JR et al. Risk of cardiovascular ischemic events after surgical castration and gonadotropin-releasing hormone agonist therapy for prostate cancer: a nationwide cohort study. J Clin Oncol. 2017;35(32):3697 3705. 10.1200/JCO.2016.71.4204) 28968166

[15] Graves JA Baig K Buntin M . The financial effects and consequences of COVID-19: a gathering storm. JAMA. 2021;326(19):1909 1910. 10.1001/jama.2021.18863) 34714325

[16] Hanna TP Evans GA Booth CM . Cancer, COVID-19 and the precautionary principle: prioritizing treatment during a global pandemic. Nat Rev Clin Oncol. 2020;17(5):268 270. 10.1038/s41571-020-0362-6) 32242095 PMC7117554

[17] Schubbe ME Gellhaus PT Tobert CM Mott SL Garje R Erickson BA . Knowledge and attitudes Regarding surgical castration in men receiving androgen deprivation therapy for metastatic prostate cancer and their relationship to health-related quality of life. Urology. 2021;155:179 185. 10.1016/j.urology.2021.04.027) 33971188

[18] Di Lorenzo G Autorino R . Androgen receptor signaling inhibitors in nonmetastatic castration-resistant prostate cancer and risk of cardiovascular toxicity: all that glitters isn't gold. Eur Urol. 2020;78(5):647 649. 10.1016/j.eururo.2020.07.036) 32800724

[19] Lawrentschuk N Fernandes K Bell D Barkin J Fleshner N . Efficacy of a second line luteinizing hormone-releasing hormone agonist after advanced prostate cancer biochemical recurrence. J Urol. 2011;185(3):848 854. 10.1016/j.juro.2010.10.055) 21239017

